# Rapid loss of an ecosystem engineer: *Sphagnum* decline in an experimentally warmed bog

**DOI:** 10.1002/ece3.5722

**Published:** 2019-10-30

**Authors:** Richard J. Norby, Joanne Childs, Paul J. Hanson, Jeffrey M. Warren

**Affiliations:** ^1^ Environmental Sciences Division and Climate Change Science Institute Oak Ridge National Laboratory Oak Ridge TN USA

**Keywords:** bog, climate change, CO_2_, moss, peatland, *Sphagnum angustifolium*, *Sphagnum fallax*, *Sphagnum magellanicum*, warming

## Abstract

*Sphagnum* mosses are keystone components of peatland ecosystems. They facilitate the accumulation of carbon in peat deposits, but climate change is predicted to expose peatland ecosystem to sustained and unprecedented warming leading to a significant release of carbon to the atmosphere. *Sphagnum* responses to climate change, and their interaction with other components of the ecosystem, will determine the future trajectory of carbon fluxes in peatlands. We measured the growth and productivity of *Sphagnum* in an ombrotrophic bog in northern Minnesota, where ten 12.8‐m‐diameter plots were exposed to a range of whole‐ecosystem (air and soil) warming treatments (+0 to +9°C) in ambient or elevated (+500 ppm) CO_2_. The experiment is unique in its spatial and temporal scale, a focus on response surface analysis encompassing the range of elevated temperature predicted to occur this century, and consideration of an effect of co‐occurring CO_2_ altering the temperature response surface. In the second year of warming, dry matter increment of *Sphagnum* increased with modest warming to a maximum at 5°C above ambient and decreased with additional warming. *Sphagnum* cover declined from close to 100% of the ground area to <50% in the warmest enclosures. After three years of warming, annual *Sphagnum* productivity declined linearly with increasing temperature (13–29 g C/m^2^ per °C warming) due to widespread desiccation and loss of *Sphagnum*. Productivity was less in elevated CO_2_ enclosures, which we attribute to increased shading by shrubs. *Sphagnum* desiccation and growth responses were associated with the effects of warming on hydrology. The rapid decline of the *Sphagnum* community with sustained warming, which appears to be irreversible, can be expected to have many follow‐on consequences to the structure and function of this and similar ecosystems, with significant feedbacks to the global carbon cycle and climate change.

## Graphical abstract

The responses of *Sphagnum* to climate change are critical to the future trajectory of peatland ecosystems and their important role in the global carbon cycle. We test three hypotheses related to the response of *Sphagnum* growth and community composition to experimental warming and CO_2_ enrichment. The decline of the *Sphagnum* community with sustained warming that we document can be expected to have many follow‐on consequences to the structure and function of this and similar ecosystems, with significant feedbacks to the global carbon cycle and climate change.

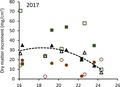

## 1 INTRODUCTION

**Figure 1:**
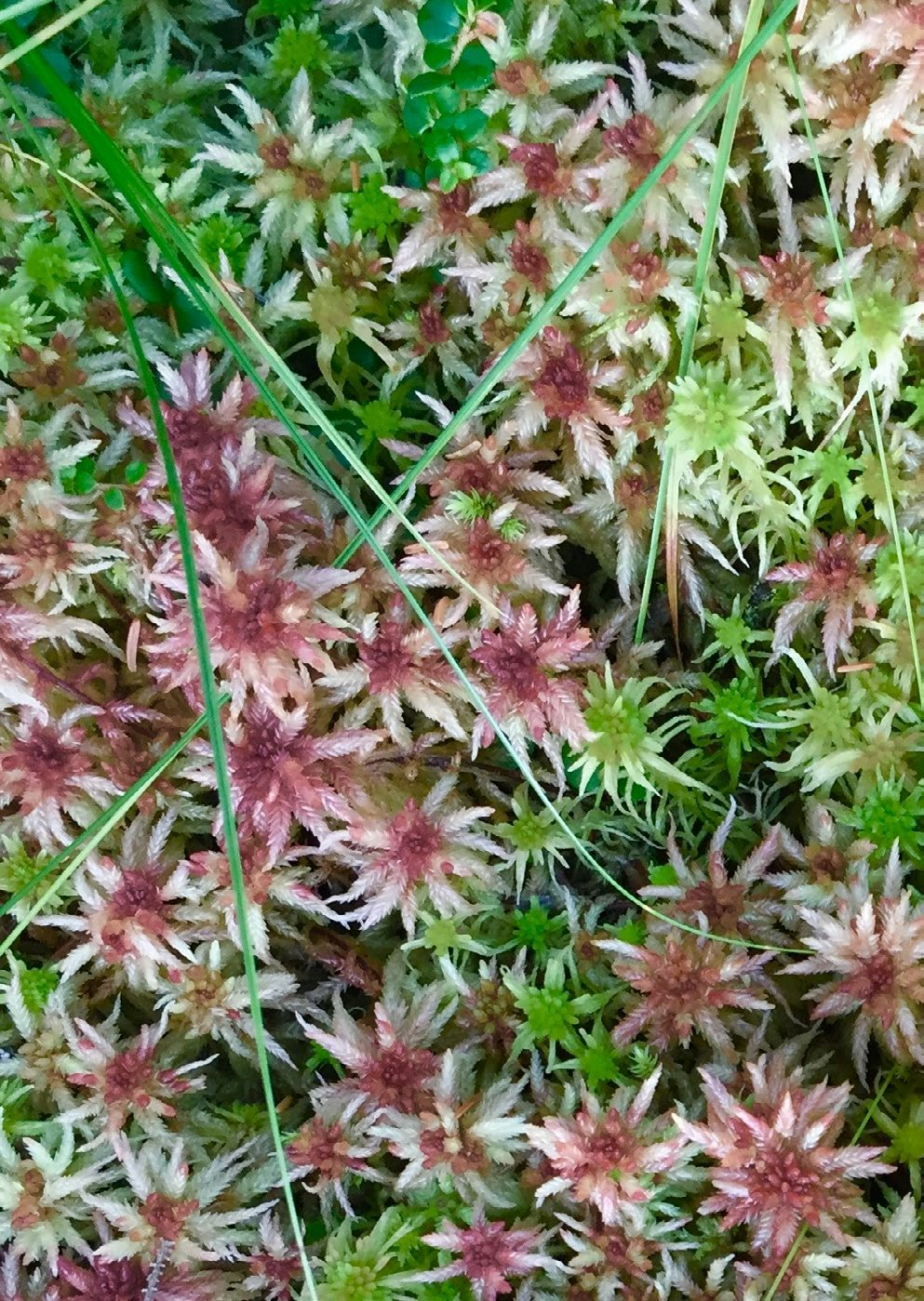
*Sphagnum magellanicum*, *S. fallax*, and *S. angustifolium* are dominant at the site of the SPRUCE experiment in northern Minnesota, USA. Photograph by Dave Weston

Boreal and subarctic peatlands contain large amounts of carbon (C), estimated to be as much as one‐fifth to one‐third of the world's soil C pool (Ciais et al., 2013; Gorham, 1991; Yu, 2012). The C accumulated in peat over centuries and millennia, because cold, acidic, and waterlogged conditions retard decomposition. Peatland C stocks are thought to be especially vulnerable to climate change because rising temperatures and associated hydrologic changes are expected to accelerate decomposition of surficial C stocks (He, He, & Hyvonen, 2016; Wilson et al., 2016), increase ecosystem respiration (Samson et al., 2018), and cause northern peatlands to become net sources of carbon to the atmosphere and exacerbating climatic warming (Gallego‐Sala et al., 2018). Hence, peatlands may be one of the most important ecosystems providing feedbacks to global climate change (Bridgham, Pastor, Dewey, Weltzin, & Updegraff, 2008; Hilbert, Roulet, & Moore, 2000; Moore, Roulet, & Waddington, 1998). The source of much of the C accumulated in peatlands is the mosses of the genus *Sphagnum* (Clymo & Hayward, 1982; Figure 1). Hence, understanding and predicting the responses of *Sphagnum* to climatic change is essential for the assessment of the responses of the peatland ecosystem and its contribution to global C budgets (Moore et al., 1998).

*Sphagnum* moss, a keystone component of boreal peatlands, is considered to be an “ecosystem engineer” that creates its own favorable conditions while forming adverse conditions for vascular plants (van Breemen, 1995; Clymo & Hayward, 1982; Granath, Strengbom, & Rydin, 2012). Its unique characteristics, including adaptations for low nutrient availability, acidification of its surroundings, and a chemical composition that retards decomposition, drive local environmental conditions that influence the presence and performance of co‐occurring plants. *Sphagnum* species are ecological specialists, sorting out in relation to pH, cation content of water, water level, and shade (Vitt & Slack, 1984). Alteration of any of those conditions brought on by atmospheric and climatic change can be expected to alter growth, vitality, or composition of the *Sphagnum* community with feedbacks to the functioning of the bog ecosystem.

We are studying *Sphagnum* responses to experimental air and soil warming in the Spruce and Peatland Responses Under Changing Environments (SPRUCE) project (Hanson et al., 2017; https://mnspruce.ornl.gov/). Located in an ombrotrophic bog in northern Minnesota, USA, at the southern edge of the boreal zone, this ecosystem is expected to be especially vulnerable to climate change. The intact ecosystem, comprising black spruce (*Picea mariana* (Mill.) B.S.P.) and tamarack (*Larix laricina* (Du Roi) K. Koch) trees, shrubs, and a nearly complete cover of *Sphagnum* mosses, is being exposed within large enclosures to a range of air and soil warming treatments in ambient or elevated atmospheric CO_2_. A comprehensive set of questions and hypotheses are being addressed on ecosystem productivity and C balance, hydrologic and nutrient cycling responses, microbial responses, and plant community ecology, all of which are closely integrated with model development. The range of attained warming of the whole ecosystem includes treatments spanning mild to very aggressive treatments (+1.6 to +10.0°C above ambient temperature), which is broader than that used in other warming experiments. The range was chosen to encompass the estimate of 7°C (with high uncertainty) for the threshold for boreal forest dieback (Lenton et al., 2008) and the projected July temperature increase for the site (~6.8°C; Peacock, 2012) under the IPCC high CO_2_ emission scenario (RCP8.5) by the year 2,100.

We structured our measurements of *Sphagnum* responses to the imposed warming and CO_2_ treatments to address the following objectives and hypotheses based on our understanding of the ecological attributes of the *Sphagnum* species at our site and observations from previous experiments.

### 1.1 Objective: Establish response surfaces of *Sphagnum* productivity response to warming

Although growth rates of *Sphagnum* spp. are sometimes positively related to moderate temperature increases (Dorrepaal, Aerts, Cornelissen, Callaghan, & van Logtestijn, 2004; Gunnarsson, 2005), such positive effects are expected to be countered by associated drying of surficial peats (i.e., the acrotelm; Robroek, Limpens, Breeuwer, & Schouten, 2007) and may not persist as the degree of warming increases (Bragazza et al., 2016).

Hypothesis: *Sphagnum* productivity will increase with modest warming and decrease as warming increases.

### 1.2 Objective: Determine whether elevated CO_2_ alters *Sphagnum* response to warming

The primary responses of vascular plants to elevated CO_2_ are an increased rate of photosynthesis and decreased stomatal aperture. *Sphagnum* photosynthesis, however, is primarily controlled by tissue water content that regulates both hydration and CO_2_ diffusion (Proctor, 2009; Schipperges & Rydin, 1998; Weston et al., 2015; Williams & Flanagan, 1996), and stomatal responses are precluded (*Sphagnum* has no stomata). While increased atmospheric CO_2_ should increase dissolved CO_2_ in the wet *Sphagnum* surface and thus CO_2_ available for photosynthesis, free‐air CO_2_ enrichment experiments in four peat bogs across Europe reported no effect of elevated CO_2_ on *Sphagnum* productivity (Hoosbeek et al., 2001).

Hypothesis: Elevated CO_2_ will have no measurable effect on *Sphagnum* growth or its response to warming.

### 1.3 Objective: Detect and quantify changes in *Sphagnum* community composition

Bryophyte species on hummocks (e.g., *S. magellanicum* Brid. and *Polytrichum strictum* Brid.), which are more distant from the water table, may be expected to be disproportionately affected by warming and the associated drying of the acrotelm (the surface layer of peat containing living plants) compared with species in the hollows (e.g., *S. fallax* (Klinggr.) Klinggr.). Alternatively, since hummock species are more adapted to drier conditions, the composition of the *Sphagnum* community may shift toward hummock species (Breeuwer, Heijmans, Robroek, & Berendse, 2008; Robroek, Limpens, Breeuwer, Crushell, & Schouten, 2007).

Hypothesis: Fractional representation of *S. magellanicum* in the *Sphagnum* community will decrease with warming and associated drying and the *S. fallax* plus *S. angustifolium* (C.E.O. Jensen ex Russow) C.E.O. Jensen fraction will increase. An alternative hypothesis is that *S. magellanicum* will be relatively favored because it is more adapted to drier conditions than the other species.

The research questions raised by these hypotheses have been tested in other experiments, but here we address them in situ at an ecosystem scale in an intact peatland system. We use a regression approach that permits us to develop response surfaces with temperature and explore whether elevated CO_2_ alters the response of *Sphagnum* to warming. Our results provide a foundation for a complete assessment of carbon cycling responses in this peatland ecosystem when integrated with other ongoing investigations of tree, shrub, and belowground responses.

## 2 MATERIALS AND METHODS

### 2.1 Study site

**Figure 2:**
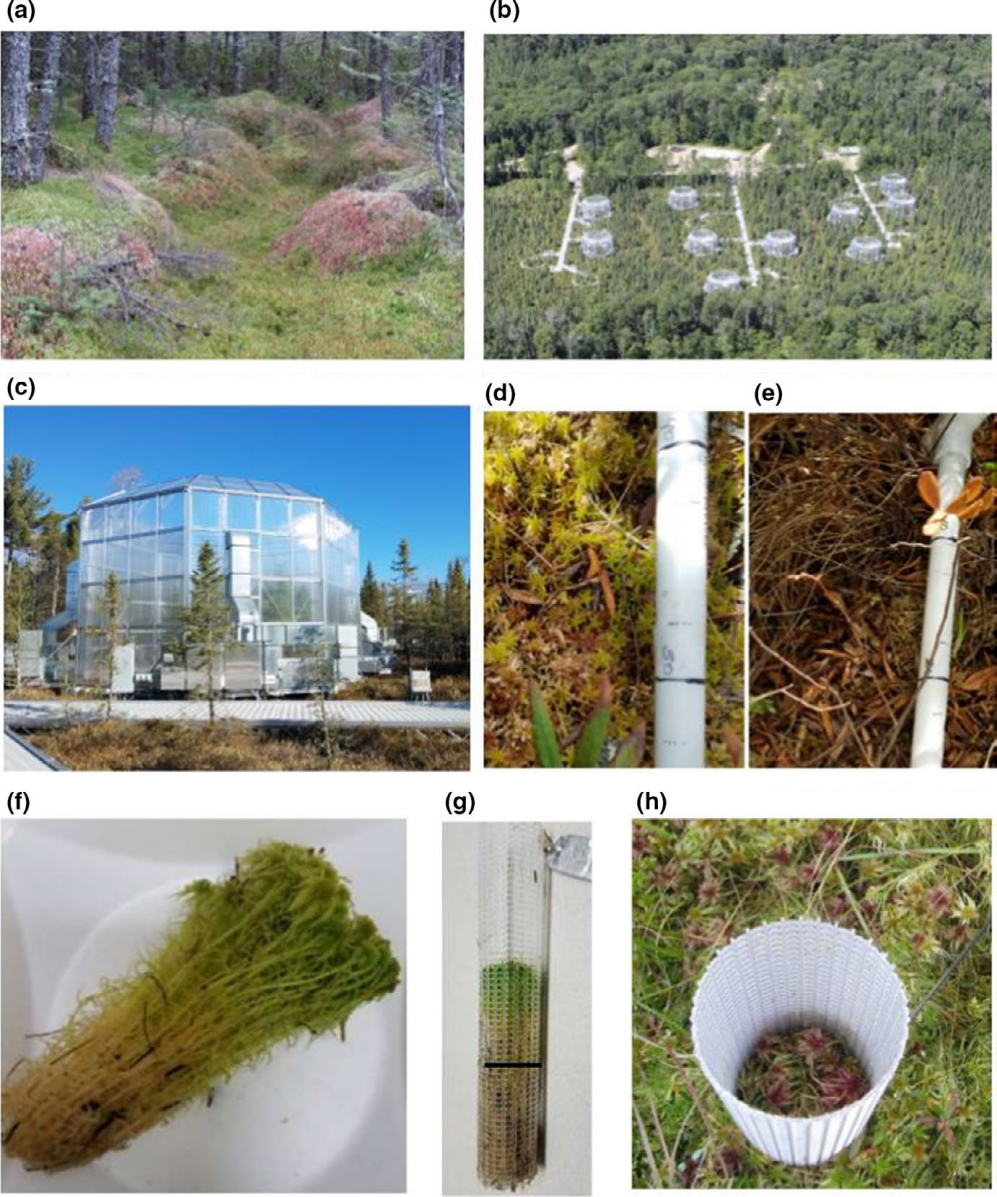
SPRUCE experiment layout. (a) Hummocks and hollows with *Sphagnum* mosses beneath *Picea mariana* trees. (b) Ten whole‐ecosystem enclosures, isolated from one another and distributed in a bog in northern Minnesota, USA. (c) Open‐top enclosure. (d, e) Guides for community composition assessments. (d) Mixture of *S. magellanicum* and *S. angustifolium*. (e) Dead *Sphagnum* beneath shrub litter. (f) Clump of *Sphagnum* cut to 7 cm length and ready for insertion into a plastic mesh column. (g) The column after removal from the field one year after deployment; the black line indicates the height of the *Sphagnum*. (h) The mesh column for measuring dry matter production inserted into the plot

The SPRUCE experiment is in an ombrotrophic peat bog in the Marcell Experimental Forest in northern Minnesota, USA (47.50283 degrees latitude, −93.48283 degrees longitude), at the southern edge of the boreal zone. Mean annual temperature (1961–2009) was 3.4°C, and average July temperature was 18.9°C, increasing 0.3°C per decade during summer months; average annual precipitation was 780 mm (Sebestyen et al., 2011). The soil is a Typic Haplohemist, with average peat depths of 2–3 m (Parsekian et al., 2012). The bog has a developing hummock and hollow microtopography (Figure 2a), with shrubs located primarily on the hummocks. The perched water table, which has little regional groundwater influence, is typically 10–20 cm above the hollows after snowmelt, receding deeper later in the growing season (Iversen et al., 2018).

As described by Hanson et al. (2017) and Griffiths et al. (2017), the bog is dominated by *Picea mariana* and *Larix laricina*, with an understory of ericaceous shrubs, including Labrador tea (*Rhododendron groenlandicum* (Oeder) Kron and Judd) and leatherleaf (*Chamaedaphne calyculata* (L.) Moench), and a limited number of herbaceous plants. Typical of bogs in northern Minnesota with an open tree cover (Vitt & Slack, 1984), there is a nearly continuous cover of mosses, primarily *Sphagnum angustifolium*, *S. fallax*, and *S. magellanicum*. [*S. magellanicum* has recently been separated into three species (Hassel et al., 2018), but the new taxonomic identity of the species in our bog has not yet been determined.] As in other similar bogs, *S. fallax* is found primarily in hollows, whereas *S. angustifolium* and *S. magellanicum* are found primarily in somewhat drier microhabitats, including lawns, low hummocks, and the flanks of high hummocks. Vitt and Slack (1984) determined *S. angustifolium* and *S. magellanicum* to have a high value of niche overlap with respect to water table height, but the niche breadth was much wider for *S. magellanicum* than for *S. angustifolium* or *S. fallax*. They note that species of the *Sphagnum* subsection Recurvum, including *S. angustifolium* and *S. fallax*, have long been a subject of controversy*. S. angustifolium* and *S. fallax* are very closely related phylogenetically, the microhabitat tolerances of the two species are superficially quite similar, and they can be difficult to distinguish in the field. In European peatlands, the two species group together in a cluster that has a high similarity in response to environmental variables (Robroek et al., 2017). At our site, *S. angustifolium* tends to dominate on hummocks and *S. fallax* in hollows, but they frequently intermix, and we have not attempted to separate them in our analyses; hence, we refer to them here as *S. angustifolium/fallax*. An initial survey of the bog prior to establishment of experimental plots indicated the following composition of the bryophyte community: 68% *S. angustifolium/fallax*, 20% *S. magellanicum*, 1% *S. fuscum*, 8% *Polytrichum strictum*, 2% *Pleurozium schreberi* (Brid.) Mitt., and scattered other species.

### 2.2 Experimental treatments

Climate change manipulations were established in 10 large, open‐top enclosures (Figure 2b,c) described by Hanson et al. (2017). The 12.8‐m diameter × 7‐m tall octagonal enclosures sheathed with double‐walled transparent greenhouse panels enable regulation of air and soil temperature and elevation of CO_2_ concentration. A subsurface corral constructed of interlocking vinyl sheet‐pile walls surrounds each plot to effect hydrologic isolation of the interior of the plot from the surrounding bog (Hanson et al., 2017; Sebestyen & Griffiths, 2016), thereby mimicking the hydrologic condition that would obtain if the entire bog was warmed to the same degree as the plot. Air warming is achieved with propane‐fired heat exchangers and a system of blowers and conduits (Hanson et al., 2017). The air warming treatments were initiated in August 2015 and were maintained 365 days per year. Target values are +0, +2.25, +4.5, +6.75, and +9°C; the +0°C enclosures are generally 1–2°C warmer than outside ambient air. Soil (peat) warming was achieved with a belowground heating array of 3‐m vertical low‐wattage heating elements installed within plastic‐coated iron pipe (Hanson et al., 2017). The combination of air and soil warming compensates for heat losses to the surrounding bog and creates a more realistic temperature profile (Hanson et al., 2011). For our analyses, warming is characterized across the active season (Richardson et al., 2018) for *Sphagnum*, here defined as the period from 15 April to 15 October. One of the two enclosures at each temperature began to receive elevated CO_2_ on 15 June 2016 to approach concentrations of 500 ppm greater than ambient, or about 900 ppm (Hanson et al., 2017).

Environmental monitoring (air and soil temperature, humidity, CO_2_ concentration) is described in Hanson, Riggs, Nettles, Krassovski, and Hook (2016, Hanson et al., 2017). Beginning in June 2018, peat water content was measured by a new method using frequency domain capacitance probes (model 10HS, Meter Group, Inc.) preinstalled into open mesh cylinders filled with 0.1 g/cm^3^ commercial peat, which were then placed laterally into the midhummock, 20 cm above the hollow position (*n* = 3). This standardized technique provided a stable relative measurement of volumetric water content (VWC) within the hummocks, whose internal structure is filled with voids and characterized by high spatial heterogeneity in bulk density. Water table height was measured with a TruTrack Water and Temperature Voltage Output sensor (Model WT‐VO 2000) within a centrally located well casing in each plot. Water table height data are referenced to the estimated mean hollow height for each plot to determine depth to water table. Raw data on water table elevation above sea level were normalized to account for variability across plots in absolute elevation, hummock heights, and proportion of hollows, thereby enabling good comparisons across plots. The normalization procedure referenced all annual data to a postsnowmelt period in late spring when water levels had drained to hollow heights (day of year 147, 158, and 150 in 2016, 2017, and 2018, respectively).

Pretreatment surveys revealed no environmental gradients in peat depth (Tfaily et al., 2014), shrub cover, or foliar nitrogen or phosphorus concentrations across the potential enclosure locations that would require repeating treatments in separate blocks. Hence, treatments were assigned to enclosures randomly. The enclosure is the experimental unit, and in all cases where multiple samples were collected from within an enclosure, the data from individual samples were pooled. Pretreatment analyses (Hanson et al., 2017) showed comparable environmental conditions within and across the constructed treatment plots. We are employing a regression‐based design to focus on the pattern of response and potential response thresholds across all warming levels and whether elevated CO_2_ alters the temperature response pattern. Regression is generally a more powerful approach than ANOVA, providing quantitative output that is more effectively incorporated into ecological models (Cottingham, Lennon, & Brown, 2005). The regression approach does not require replicating levels of warming, and indeed, if more plots had been available, they would have been assigned to intermediate levels of warming rather than replications of existing levels. Although we cannot ask questions about any differences in response between individual enclosures, such questions are not relevant to our overall objectives.

### 2.3 Community composition

To monitor changes in the species composition of the bryophyte community, we established three transects within each plot, embedded within 1 m × 2 m plant community assessment areas where no destructive sampling or instrumentation was permitted. There are 5–10 permanently located 5 cm × 5 cm sample points in each transect, 25 total in each experimental plot. Although the total area surveyed is small, the linear extent of the transects is approximately 28% of the diameter of the measurable plot area. Permanent stakes mark the endpoints of the transects; we attach a removable, calibrated plastic tube to the stakes to ensure that we can locate the same sample points each year (Figure 2d,e; additional photographs and maps of these transects can be seen in the data archive (Norby & Childs, 2018)). We assessed the community composition in October 2015 (prior to initiation of the whole‐ecosystem warming and CO_2_ treatments), 2016, 2017, and 2018. At each assessment point, we estimated the percentage coverage by *S. angustifolium/fallax*, *S. magellanicum*, *Polytrichum strictum*, *Pleurozium schreberi*, or (rarely) other *Sphagnum* species or lichen. We visually estimated cover of each component to the nearest 10% and averaged over the 25 spots. We also noted the percentage of the area that was bare or covered with dead moss. Each spot was coded as whether the location was hummock or hollow after measuring the distance to a level reference marker and the vertical distance from the reference line to the bottom of a nearby hollow, with sample points at heights 10 cm or more above the deepest nearby hollow considered on a hummock. All of the observations over the four years were made by the same observer. Evaluations over larger areas or from overhead photographs were precluded because of the shrub cover; it was necessary for the observer's eyes to be quite close to the surface for accurate assessments. To test the accuracy of our fractional cover estimates, we surveyed a similar 1‐m transect outside of an enclosure and then harvested the ten 5 cm × 5 cm sample points and measured the area of *S. angustifolium/fallax*, *S. magellanicum*, and *Polytrichum* in the laboratory. Correlation coefficients were 0.66–0.70, and the averages of the field estimates of the 10 samples points were 6.5% low for *S. angustifolium/fallax*, 4.6% high for *S. magellanicum*, and 1.9% high for *Polytrichum*.

### 2.4 *Sphagnum* growth

In preliminary measurements (Griffiths et al., 2017), we realized that common methods for measuring *Sphagnum* growth were not effective at this site. Crank or brush wires (Clymo, 1970; Rydin & Jeglum, 2013), which measure increases in stem length along a vertical wire, could not capture growth of *Sphagnum* in hollows, which were only rarely vertically oriented, and they could not be used over winter to capture growth immediately after snowmelt in spring. Bundles of 5‐cm‐long *Sphagnum* stems secured with string and reinserted into the bog (Grosvernier, Matthey, & Buttler, 1997) could be used over winter, but retrieval in the fall was often compromised, especially when there was substantial growth. Furthermore, both of these methods have multiple sources of error when attempting to scale production to grams per square meter, which requires data on increase in length, mass per unit length, and number of stems per unit area.

In light of these difficulties, we developed a more direct approach. We measured *Sphagnum* growth (or dry matter increment) in mesh columns, constructed from rigid polypropylene mesh tubes (Industrial Netting, RN1500) (Figure 2g; additional photographs in the data archive (Norby & Childs, 2018)). The tubes have an inside diameter of 38 mm, with a wall thickness of 1.5 mm and 46% open area. We cut them to 22 cm length and attached a disk of mesh material to the bottom using small plastic cable ties. We initiated the annual growth assessment period in October after growth had ended for the year while ensuring the columns were in place to capture spring growth in the next year. We harvested clumps of *Sphagnum* from each plot, removed debris, and cut the stems to 7 cm length (Figure 2f). We set up measurements for three types of *Sphagnum*: *S. angustifolium/fallax* from hummocks, *S. angustifolium/fallax* from hollows, and *S. magellanicum* from hummocks. We prepared two sets of the three types of *Sphagnum* by inserting the *Sphagnum* into the columns at a stem density similar to that of the bog (Figure 2g), measuring the distance from top of the column to top of the *Sphagnum*, and installing the columns into the bog, ensuring that the top of the *Sphagnum* in the column was even with and closely coupled to the surrounding *Sphagnum* (Figure 2h). We installed two sets of three columns in different locations in the plots, each set comprising a column with *S. angustifolium/fallax* and a column with *S. magellanicum* on a hummock and a *S. angustifolium/fallax* column in an adjacent hollow. Additional photographs and description of this procedure are in the data archive (Norby & Childs, 2018).

In May, after snowmelt and thaw of the acrotelm, we adjusted the position of the column in the bog when occasionally necessary to maintain the *Sphagnum* in the column at the same height as the *Sphagnum* capitula surrounding the column. We repeated the measurement of column top to *Sphagnum* top in May and again at the end of the growth assessment period in October. We removed the columns from the bog in October, separated new *Sphagnum* growth (i.e., tissue above the initial 7 cm stem), and obtained the fresh and dry mass. We inserted new columns for the next year's assessment at the same locations. Annual growth is reported as dry matter increment (DMI) in milligrams per square centimeter of bog surface. The growth assessments were based on new *Sphagnum* stems each year, so cumulative effects of warming were largely precluded. The columns were deployed amidst healthy *Sphagnum* patches even when other areas of the plot exhibited desiccation and *Sphagnum* loss, so these measurements represent the growth potential. The fraction of growth that occurred in spring was determined as the ratio of length increment from October to May (with the assumption that all this growth occurred in spring) to total annual length increment.

### 2.5 Net primary production (NPP)

We calculated NPP at the scale of the whole plot as the sum of dry matter increment of the three *Sphagnum* type times its respective fractional coverage times the mean *Sphagnum* tissue C fraction of 0.429; the data are reported in grams C per square meter per year. Carbon fraction was determined on multiple collections of *Sphagnum* from the site using a Model 4010 Elemental Combustion System (Costech Analytical Technologies).

### 2.6 Data analysis

We evaluated the effects of temperature on *Sphagnum* DMI, NPP, and community composition data by stepwise multiple linear regression analysis (Statistix, Ver. 8.0), with air temperature (*T*), *T* ^2^ (to address possible curvilinear responses), [CO_2_], and [CO_2_] × temperature as possible factors. A *p* value of .05 was required for a factor to enter the model. For the two instances in which both [CO_2_] and temperature effects are significant, we evaluated separate regressions against temperature for the two CO_2_ treatments, and a significant CO_2_ × temperature indicates different slopes. When only temperature effects are significant, we present regressions using all 10 enclosures. Although we sometimes refer to the different enclosures by their target treatment values (+0, +2.25, +4.5, +6.75, and +9°C), in the regression analysis we used the actual temperature measured at 0.5 m above the hollows and averaged over the period 15 April to 15 October (Table 1). We used air temperature above the hummocks in these analyses because it was the metric previously used in models of *Sphagnum* gross primary production (Walker et al. 2017). To evaluate possible mechanisms by which air temperature affected *Sphagnum* growth and NPP, we conducted additional analyses in which NPP in 2018 was regressed against VWC of hummocks, maximum depth to water table, and soil temperature at 20 cm depth. Vapor pressure deficit (VPD), calculated from measured temperature and relative humidity, was linearly related to air temperature (correlation coefficient = 0.988); therefore, VPD provided no additional information as a regressor. Data used in these analyses are freely available (Norby & Childs, 2018).

**Table 1:** Environmental conditions over the period from 15 April to 15 October in the experimental enclosures and ambient air in 2016–2018

Year	Plot#	CO_2_ treatment	Nominal warming treatment	Average air temperature (^o^C)	Differential from ambient (^o^C)	Relative humidity (%)	Vapor pressure deficit (kPa)	CO_2_ (ppm)	Average soil temperature at 5 cm (^o^C)	Maximum depth to water table (cm)	VWC of hummock (m^3^/m^3^)
2016	Open	Ambient	Ambient	15.78	–	79.2	0.53	404	11.95	9.30	–
2016	6	Ambient	+0	17.57	1.79	76.1	0.66	404	14.02	8.03	–
2016	19	Elevated	+0	17.49	1.72	75.8	0.66	731	14.23	5.77	–
2016	20	Ambient	+2.25	20.26	4.48	61.6	1.11	403	14.21	11.94	–
2016	11	Elevated	+2.25	20.10	4.33	62.6	1.08	705	15.16	6.20	–
2016	13	Ambient	+4.5	22.03	6.25	56.3	1.38	411	15.29	12.61	–
2016	4	Elevated	+4.5	21.97	6.20	58.0	1.34	731	16.12	10.86	–
2016	8	Ambient	+6.75	23.65	7.88	52.8	1.59	399	17.14	22.73	–
2016	16	Elevated	+6.75	23.88	8.10	50.1	1.71	720	17.39	20.42	–
2016	17	Ambient	+9	24.90	9.12	47.4	1.87	419	16.21	24.42	–
2016	10	Elevated	+9	25.78	10.01	44.2	2.07	701	11.56	18.47	–
2017	Open	Ambient	Ambient	14.52	–	79.8	0.50	406	11.11	17.07	–
2017	6	Ambient	+0	16.32	1.80	76.9	0.63	409	10.15	15.36	–
2017	19	Elevated	+0	16.13	1.61	76.5	0.62	849	13.17	14.15	–
2017	20	Ambient	+2.25	19.32	4.80	62.3	1.06	407	13.60	21.58	–
2017	11	Elevated	+2.25	18.64	4.12	63.8	0.98	849	12.98	12.29	–
2017	13	Ambient	+4.5	20.80	6.28	57.5	1.28	412	14.11	27.54	–
2017	4	Elevated	+4.5	20.31	5.79	60.7	1.19	884	15.03	18.51	–
2017	8	Ambient	+6.75	22.34	7.82	52.6	1.50	412	15.37	31.70	–
2017	16	Elevated	+6.75	22.65	8.13	50.0	1.62	872	16.01	17.87	–
2017	17	Ambient	+9	23.62	9.10	47.0	1.78	415	17.24	32.11	–
2017	10	Elevated	+9	24.35	9.83	45.6	1.89	852	16.90	35.87	–
2018	Open	Ambient	Ambient	14.96	–	79.6	0.52	409	11.09	16.12	0.603
2018	6	Ambient	+0	17.21	2.24	73.6	0.97	423	9.71	16.97	0.575
2018	19	Elevated	+0	16.77	1.80	62.2	1.40	708	11.39	27.90	0.547
2018	20	Ambient	+2.25	19.47	4.51	55.3	1.84	415	12.23	18.14	0.589
2018	11	Elevated	+2.25	19.04	4.07	49.1	2.26	698	12.33	26.65	0.543
2018	13	Ambient	+4.5	21.68	6.72	45.5	2.52	415	13.23	32.05	0.532
2018	4	Elevated	+4.5	21.27	6.31	73.0	0.94	723	14.63	23.06	0.534
2018	8	Ambient	+6.75	23.46	8.49	60.5	1.40	416	15.30	37.35	0.520
2018	16	Elevated	+6.75	23.31	8.34	57.7	1.78	743	16.43	24.86	0.513
2018	17	Ambient	+9	24.44	9.47	46.8	2.22	429	16.57	43.96	0.460
2018	10	Elevated	+9	25.28	10.32	44.9	2.65	736	16.17	44.99	0.455

Note

Air temperature and relative humidity were measured 0.5 m above the level of hollows; vapor pressure deficit was calculated from temperature and relative humidity. CO_2_ concentration was measured at 0.5 m height; values are the daytime (06:00–18:00 hr) averages from 15 April to 15 October. Soil temperature was measured at 5 cm depth. Depth to water table relative to the level of hollows was measured in 2016–2017 with water column sensors. The maximum values occurred in late summer (mid‐August to mid‐September). Volumetric water content (VWC) of hummocks was measured beginning in 2016, but effective methods applicable to the peatland hummock‐hollow topography were only viable from 3 July to 7 October 2018; average values over the time period are shown.

## 3 RESULTS

### 3.1 *Sphagnum* community composition

**Figure 3:**
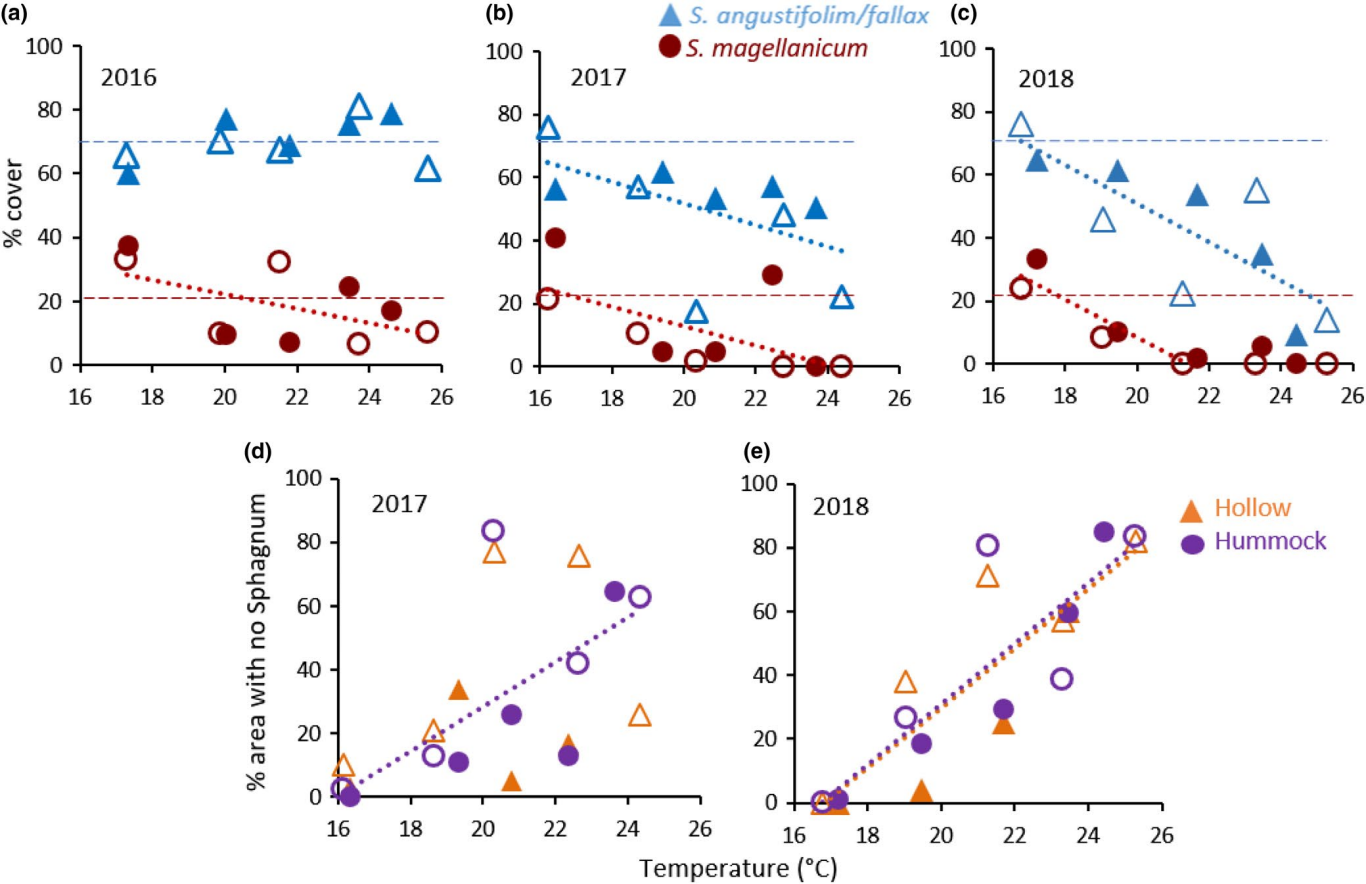
Fractional cover of *Sphagnum*. (a, b, c) Fractional cover of *S. magellanicum* (red circles) and *S. angustifolium/fallax* (blue triangles) plotted against average air temperature (*T*) measured at 0.5 m above hollow surface from 15 April to 15 October. Solid symbols are ambient CO_2_; open symbols are elevated CO_2_. Horizontal dashed lines represent the pretreatment (2015) fractional cover. (a) 2016: regression of *S. magellanicum* against temperature: %cover = –2.27 × *T* + 67.73, *r* ^2^ = .32, *p* = .09. (b) 2017: regression of *S. magellanicum* cover against temperature: %cover = –3.07 × *T *+ 74.01, *r* ^2^ = .38, *p* = .06; *S. angustifolium/fallax*: %cover = –3.46 × *T* + 120.80, *r* ^2^ = .32, *p* = .09. (c) 2018: regression of *S. magellanicum* cover against temperature, excluding +6.75 and +9°C enclosures: %cover = –5.98 × *T* + 127.96, *r* ^2^ = .85, *p* = .006; *S. angustifolium/fallax*: %cover = –6.10 × *T* + 173.11, *r* ^2^ = .64, *p* = .005. (d, e) Fraction of ground area with no live *Sphagnum* in hollows (orange triangles) or on hummocks (purple circles). Solid symbols are ambient CO_2_; symbols are elevated CO_2_. (d) 2017: regression for hummocks: %area = 6.97 × *T* − 110.86, *r* ^2^ = .46, *p* = .03. (e) 2018: regression for hollows: %area = 9.39 × *T* − 158.21, *r* ^2^ = .75, *p* = .003; hummocks: %area = 9.48 × *T* − 158.68, *r* ^2^ = .74, *p* = .001

In October 2015, prior to initiation of the warming and CO_2_ treatments, 70.1 ± 2.9% of the ground area within the experimental enclosures was covered by *S. angustifolium* or *S. fallax* and 20.3 ± 3.6% by *S. magellanicum*; *Polytrichum strictum*, *Pleurozium schreberi*, and other mosses or herbaceous vascular plants each covered less than 5% (Table 2). Cover of *S. magellanicum* tended to decline with increasing temperature in 2016 after treatments commenced (Figure 3a), but there was no detectable change in *S. angustifolium/fallax* or total *Sphagnum* cover. An effect of warming was apparent in 2017 (Figure 3b). *Sphagnum* cover decreased with increasing temperature, declining from 97% in the two +0°C plots to 22 and 50% in the two +9°C plots.

**Table 2:** Pretreatment (2015) composition of the moss community

Plot#	Future CO_2_ treatment	Future warming treatment	*S. angustifolium/fallax*	*S. magellanicum*	*Polytrichum strictum*	*Pleurozium schreberi*
% cover						
6	Ambient	+0	56.8	42.0	0.4	0.0
19	Elevated	+0	67.6	28.4	0.0	0.0
20	Ambient	+2.25	80.0	4.8	3.2	8.0
11	Elevated	+2.25	80.0	14.8	0.8	0.4
13	Ambient	+4.5	68.0	6.0	2.0	21.6
4	Elevated	+4.5	56.4	39.6	0.0	0.0
8	Ambient	+6.75	71.2	28.8	0.0	0.0
16	Elevated	+6.75	86.7	13.3	0.0	0.0
17	Ambient	+9	78.4	16.4	1.2	0.0
10	Elevated	+9	59.2	9.6	25.2	2.0
Average			70.1	20.3	4.2	2.7

Both *S. angustifolium/fallax* and *S. magellanicum* declined with increasing temperature. Fractional coverage of *S. angustifolium/fallax* declined to 36.2% in the +9°C plots; fractional coverage of *S. magellanicum* was just 3.3% in +4.5°C enclosures, and there was no *S. magellanicum* remaining in any of the sample locations in +9°C enclosures. Casual observations of 1.13‐m^2^ subplots from which shrubs had been removed confirmed these trends. Although the slopes of the declines in percent cover with increasing temperature were similar (3.1 and 3.5 per °C), the relative loss of *S. magellanicum* relative to its cover in +0 enclosures was greater than the relative loss of *S. angustifolium/fallax* (10% vs. 5%). The loss of *Sphagnum* cover continued in 2018 (Figure 3c), and the slopes of the declines were twice that in 2017. The relative decline in *S. magellanicum* cover was especially severe, resulting in its complete absence at any of the observation points in the two +9°C enclosures. Cover of *Polytrichum* or other mosses did not change with time or treatment.

These declines were due to the increase in bare ground or dead *Sphagnum* in 2017 and 2018 (Figures 2e and 3d,e). Less than 3% of the ground had no live moss in 2015, 2016, or the +0 enclosures in 2017, but non‐*Sphagnum* area on hummocks increased with temperature in 2017 and averaged 63% in the +9°C enclosures. Bare ground also occurred in hollows; there was no consistent trend with temperature in 2017 (Figure 3d), but non‐*Sphagnum* area increased with temperature similarly in both hummocks and hollows in 2018 (Figure 3e). Eighty five percent of the ground area in +9°C enclosures had no live moss. The desiccation and loss of the *Sphagnum* community appears to be irreversible as we saw no sign of recovery during the cooler, wetter days of early spring.

### 3.2 Growth

**Figure 4:**
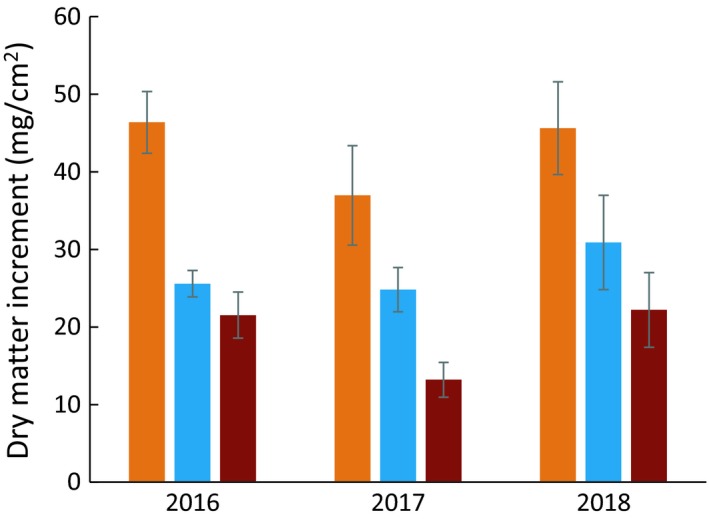
Annual dry matter increment (DMI) of *S. angustifolium/fallax* in hollows (orange), *S. angustifolium/fallax* on hummocks (blue), and *S. magellanicum* on hummocks (red), averaged across all levels of warming and CO_2_ treatments. Error bars represent ±1 *SE* (*n* = 10)

**Figure 5:**
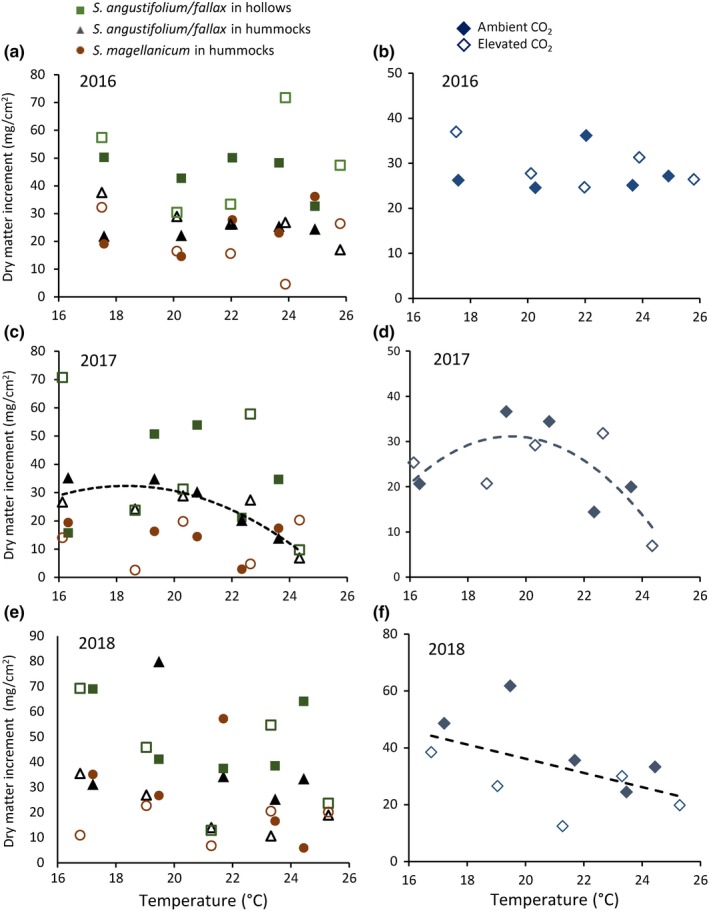
Dry matter increment of *Sphagnum* relative to average air temperature (*T*) measured at 0.5 m above hollow surface from 15 April to 15 October. (a, c, e) Responses in 2016, 2017, and 2018 of *S. angustifolium/fallax* in hollows (green squares), *S. angustifolium/fallax* on hummocks (black triangles), and *S. magellanicum* on hummocks (red circles). Solid symbols are ambient CO_2_; open symbols are elevated CO_2_. (b, d, f) Average of data in (a, c, and e), weighted by fraction of total *Sphagnum* cover. Solid diamonds are ambient CO_2_; open diamonds are elevated CO_2_. (c) Regression of *S. angustifolium/fallax* on hummocks against temperature in 2017: DMI = 231.8 × *T* − 6.32 × *T* ^2^ − 1,800, *r* ^2^ = .76, *p* = .03. (d) Regression of weighted average growth against temperature in 2017: DMI = 334.3 × *T* − 8.57 × *T* ^2^ − 2,948, *r* ^2^ = .52, *p* = .04. (f) Regression of weighted average growth against temperature in 2018: DMI = –2.42 × *T* − 0.050 × [CO_2_] + 112.80 (*r* ^2^ = .59, *p* = .05). Although [CO_2_] was a significant factor, regressions within ambient or elevated CO_2_ were not significant

Averaged across all enclosures, *Sphagnum* growth (annual DMI) was greater in hollows than in hummocks (Figure 4). Our results (after multiplying by 10 to convert to g/m^2^) are consistent with reported mean growth rates of these species: *S. fallax* (primarily in hollows), 400 g m^−2^ year^−1^; *S. angustifolium* (primarily on hummocks), 180 g m^−2 ^year^−1^; and *S. magellanicum*, 250 g m^−2 ^year^−1^ (Gunnarsson, 2005). Approximately 30% of annual growth occurred in early spring of 2017 and 12% in 2018, but there were wide variation and no pattern with respect to species or treatment (data presented in Norby & Childs, 2018). There were no effects of temperature or CO_2_ on DMI in 2016 (Figure 5a,b), but *S. angustifolium/fallax* exhibited a curvilinear relationship with temperature in 2017 (Figure 5c), which was reflected in the cover‐weighted average DMI (Figure 5d). Maximum growth occurred at 19.5°C, or 5.0°C above growing season ambient temperature. However, increased growth with modest warming did not persist in 2018. Although there were no clear trends in any of the three *Sphagnum* groups (Figure 5e), cover‐weighted average DMI declined linearly with increasing temperature in 2018, and DMI was less in elevated CO_2_ (Figure 5f).

### 3.3 Plot‐level production

**Figure 6:**
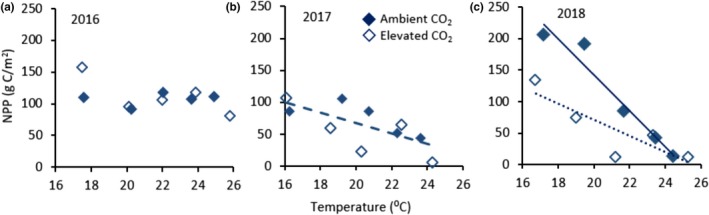
Plot‐level net primary productivity (NPP) relative to average air temperature (*T*) measured at 0.5 m above hollow surface from 15 April to 15 October, plotted as in Figure 5. (a), 2016, no effects of temperature or [CO_2_]. (b) 2017, regression of NPP against temperature: NPP = –8.12 × *T* + 229.6, *r* ^2^ = .50, *p* = .02. (c) 2018, NPP = –53.0 × *T* − 1.40 × [CO_2_] + 0.057 × (*T* * CO_2_) + 1,309, *r* ^2^ = .92, *p* = .03. Ambient CO_2_: NPP = –28.64 × *T* + 715.83, *r* ^2^ = .95, *p* = .005; elevated CO_2_: NPP = –12.81 × *T* + 326.7, *r* ^2 ^= .74, *p* = .06

**Figure 7:**
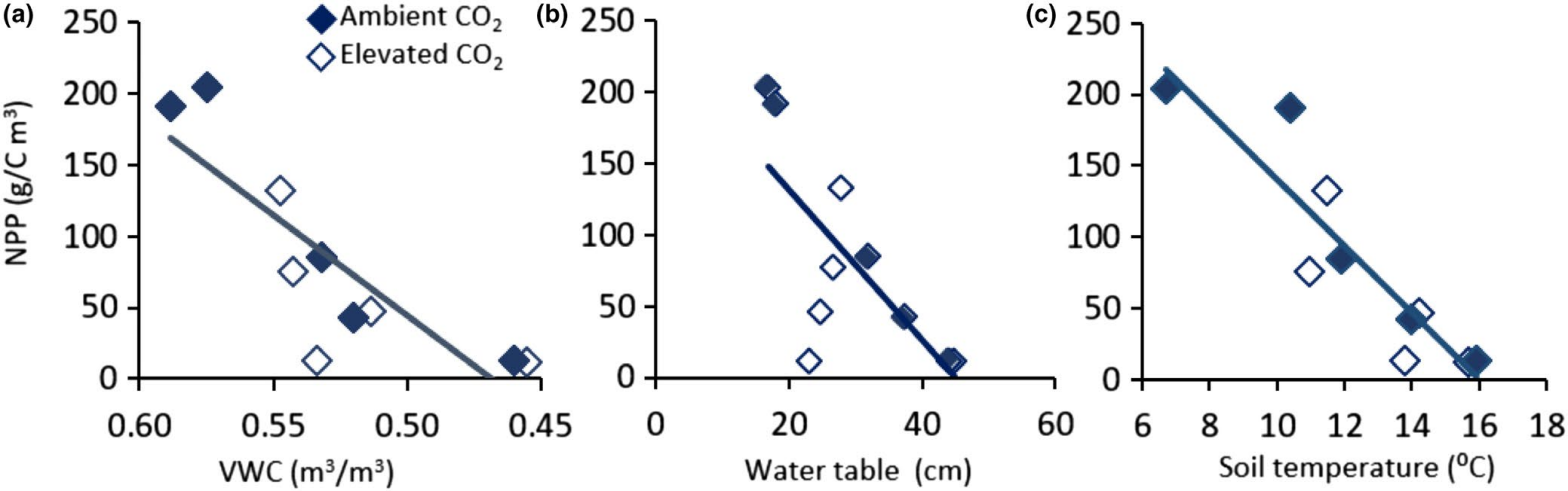
Plot‐level net primary productivity (NPP) in 2018 relative to secondary factors that changed with warming. (a) NPP in relation to the average volumetric water content (VWC) of hummocks from 3 July to 7 October 2018. Note that the x‐axis is displayed in increasing dryness (decreasing VWC) since VWC declined with increasing temperature (Table 1). NPP = 1,404 × VWC − 657.8, *r* ^2^ = .71, *p* = .002. (b) NPP in relation to maximum depth to the water table WT. NPP = −5.26 × WT + 237.1, *r* ^2^ = .52, *p* = .02. (c) NPP relative to average soil temperature at 20 cm depth from 15 April to 15 October. NPP = −26.66 × Tsoil + 449.5, *r* ^2^ = .77, *p* = .001. [CO_2_] and the interaction with [CO_2_] were not significant in any of these relationships

There was no effect of temperature on NPP (DMI × fractional cover) in 2016 (Figure 6a), but warming had a large effect on *Sphagnum* NPP in the second and third years after the onset of treatments (Figure 6b,c) due primarily to the loss of cover. In 2018, we also observed a negative effect of CO_2_ enrichment on NPP, and there was a significant CO_2_ × temperature interaction, indicating that the slopes of NPP versus temperature were different in the two CO_2_ levels. The slope of the linear regressions indicates a loss of 8 g C/m^2^ of NPP per degree temperature increase in 2017. The loss in NPP was greater in 2018:29 and 13 g C/m^2^ of NPP per degree temperature in ambient and elevated CO_2_, respectively. The effect of temperature on NPP was related to the water status of the bog. The maximum depth to the water table increased, and VWC of hummocks decreased with warming (Table 1). NPP in 2018 declined in relation to both VWC and water table depth, with no effect of CO_2_ (Figure 7a,b). NPP also declined with increasing soil temperature (Figure 7c).

## 4 DISCUSSION

An important attribute of the regression‐based design we employed in this experiment has been our ability to describe the response surface of *Sphagnum* in an intact ecosystem to increased air and soil temperatures that encompass the range of warming that is predicted to occur over this century. We anticipated that warming of this bog ecosystem would cause a loss of *Sphagnum* productivity and that the loss in productivity would be driven primarily by the effects of temperature on water status. That expectation was realized, and the effects of warming were large, driven by a wide‐scale desiccation and loss of the *Sphagnum* community with increasing temperature and with effects increasing over time as a cumulative response. Reduction in growth potential also contributed to the loss in productivity. Recognizing that some studies have shown increases in *Sphagnum* growth with warming (Dorrepaal et al., 2004; Robroek, Limpens, Breeuwer, & Schouten, 2007), our working hypothesis was that there would be a curvilinear response. This hypothesis was supported in 2017: DMI of *Sphagnum* increased with modest warming and then decreased with more aggressive warming, with the maximum growth occurring when average temperature was 5.0°C greater than the growing season average ambient temperature. The positive response to warming is consistent with the earlier onset of spring observed in the warmer enclosures (Richardson et al., 2018), a mechanism that would have been precluded in most other experiments that do not maintain continuous, multiyear warming treatments. However, all other responses we observed in this experiment were linear, and our hypothesis of a curvilinear response is rejected.

Other experimental studies with more modest warming treatments have produced mixed results. Summer warming (+0.9°C) in open‐top chambers in Abisko, Sweden, enhanced biomass production of *Sphagnum fuscum* (Dorrepaal et al., 2004). Biomass production of *S. magellanicum* and *S. rubellum* from a bog in Ireland was greater at 20°C than at the average site temperature of 15°C but was reduced when the water table was lowered (Robroek, Limpens, Breeuwer, Crushell, et al., 2007). Lichen and bryophyte cover decreased in response to 1–3°C warming at 11 tundra sites, but there was no significant effect on *Sphagnum* (Walker et al., 2006). Increased soil temperature (1.7–4.5°C at 15 cm depth) did not affect bryophyte production in monoliths from a Minnesota bog, but production was driven strongly by water table depth (Weltzin, Harth, Bridgham, Pastor, & Vonderharr, 2001). Increased air temperature (+3.6°C) and associated increased evapotranspiration significantly reduced *Sphagnum* growth independent of water table in a poor fen in Sweden (Gunnarsson, Granberg, & Nilsson, 2004). *S. fallax* productivity decreased by 60% in mesocosms transplanted to a warmer (+5°C) location (Bragazza et al., 2016). Maximum photosynthesis of several *Sphagnum* species occurred at 30–35°C, but these observations were made on fully water‐saturated *Sphagnum* stems in the laboratory (Haraguchi & Yamada, 2011); most temperate species have photosynthetic optima between 15 and 25°C (He et al., 2016). Relevant insights into *Sphagnum* responses under natural conditions emerged from the record‐breaking 2003 heat wave across Europe, which was associated with widespread die‐off of *Sphagnum* (Bragazza, 2008); hummocks in 20 bogs Italian Alps had irreversibly desiccated *Sphagnum* with no sign of recovery 4 years later. *Sphagnum* forming lawns and carpets also desiccated but recovered in subsequent years. Similarly, in our experiment there has been no indication of recovery of the *Sphagnum* community. Although we have not relaxed the treatments, the exposure temperatures follow a seasonal course, and recovery was not observed during the cooler, wetter conditions in spring in either hummocks or hollows.

Ecosystem warming can have multiple direct and indirect effects on ecosystem processes, and it can be difficult to tease the different effects apart. The decline in *Sphagnum* growth and NPP in this experiment might have been a direct result of effects on physiological processes in *Sphagnum* (Schipperges & Rydin, 1998; Van Gaalen, Flanagan, & Peddle, 2007; Walker et al., 2017) or indirect effects from changes in competition with shrubs for light or nutrients (He et al., 2016; Malmer, Svensson, & Wallen, 1994; Turetsky et al., 2012). Increased mineralization of peat, as would likely occur with warming and lowering of the water table, has been shown to increase growth of vascular plants in peatland ecosystems, leading to increased shading and reduced growth of intolerant *Sphagnum* (Malmer et al., 1994). The most likely or most dominant mechanism of response, however, was probably through the effect of warming on depth to the water table and water content of the acrotelm (Grosvernier et al., 1997; Rydin, 1985; Weltzin et al., 2001), both of which responded to increasing temperature (Table 1). NPP in 2018 was negatively correlated with the greater depth to the water table and reduced VWC measured in the hummocks (Figure 7a,b). A low water table can reduce *Sphagnum* growth by reducing the capillary rise of water to the capitula (Goetz & Price, 2016). Desiccation of capitula due to increased evaporation associated with higher temperatures and vapor pressure deficits can reduce *Sphagnum* growth independent of the water table depth (Gunnarsson et al., 2004).

We saw no growth stimulation of *Sphagnum* in elevated CO_2_, consistent with previous observations and in support of our hypothesis for this experiment. Mini‐FACE experiments in four predominantly ombrotrophic peat bogs in Finland, Sweden, the Netherlands, and Switzerland showed no effect of elevated CO_2_ on *Sphagnum* biomass over 3 years (Hoosbeek et al., 2001). In a greenhouse experiment, *Sphagnum* growth was initially stimulated by elevated CO_2_, but the response did not persist in the second year of exposure, and in the field, *Sphagnum* responded more to spatial variation in hydrology than to atmospheric CO_2_ concentrations (Toet et al., 2006). In contrast with vascular plants for which effects of elevated CO_2_ on photosynthesis and stomatal conductance are well documented (Ainsworth & Long, 2005), *Sphagnum* photosynthesis is controlled more by water content (Schipperges & Rydin, 1998; Williams & Flanagan, 1996), and stomatal responses are precluded. Furthermore, methane can be a significant C source for submerged *Sphagnum* (Raghoebarsing et al., 2005); refixation of CO_2_ derived from decomposition processes also is an important source of C for *Sphagnum* (Rydin & Clymo, 1989; Turetsky & Wieder, 1999). The reliance on substrate‐derived CO_2_, especially in wet conditions when there is increased resistance to CO_2_ diffusion from the atmosphere, can explain the lack of response of *Sphagnum* to atmospheric CO_2_ enrichment (Smolders, Tomassen, Pijnappel, Lamers, & Roelofs, 2001). There were, however, significant negative effects of elevated CO_2_ on DMI and NPP in 2018, which we assume was an indirect effect of CO_2_ stimulation of shrub or tree growth, increasing shading of *Sphagnum* or altering water balance. *S. magellanicum* in particular has a narrow niche breadth with respect to shade (Vitt & Slack, 1984). Measurements of leaf area index and normalized difference vegetation index (NDVI) documented increased shrub cover in warmer enclosures and effects of elevated CO_2_ on NDVI only in the warmest enclosures (McPartland et al., 2019). Negative effects of elevated CO_2_ on *Sphagnum* growth also were reported in a greenhouse study with peat monoliths (Heijmans, Klees, de Visser, & Berendse, 2002). They attributed the response to an interaction between CO_2_ and the higher greenhouse temperature compared with outdoors, and possibly related to an accumulation of allelopathic substances, but not associated with changes in vascular plant cover. The negative effect of elevated CO_2_ on *Sphagnum* we observed in this experiment is surprising and emphasizes the importance of a whole‐ecosystem analysis. Ongoing studies will focus on revealing the interactions between *Sphagnum* and other ecosystem components, particularly the interactions with shrubs and with nitrogen availability.

Our third hypothesis posited that *Sphagnum* species on the hummocks, which are further from the water table, would be disproportionately affected by the warming treatments compared with *Sphagnum* in the wetter hollows. An alternate hypothesis would suggest that *Sphagnum* species that are more adapted to the wetter conditions of the hollows would be impacted more. The decline in *Sphagnum* cover affected both *S. angustifolium/fallax* and *S. magellanicum*, but the relative loss of *S. magellanicum* was greater, in support of the primary hypothesis. *S. magellanicum*, which was present primarily on hummocks and has morphological adaptations generally favorable for drier conditions, comprised a smaller fraction of the *Sphagnum* community in enclosures exposed to the warmer temperatures. However, the dominant response was a sharp decline in abundance of both species, and there was no evidence that *S. angustifolium/fallax* was replacing *S. magellanicum*. There was no support for the alternative hypothesis that species more adapted to dry conditions (e.g., *S. magellanicum* and *Polytrichum*) would be more resistant to the stress and would increase in dominance. Other *Sphagnum* species that are often associated with dry hummocks (e.g., *S. fuscum* and *S. capillifolium*) occur on our site but with insufficient abundance (<1% cover) for evaluating whether these species would respond substantially differently from *S. magellanicum*. Our results contrast with observations in a *Sphagnum*‐dominated bog in the Jura Mountains of France, where warming of 1.3°C caused a decrease in moss abundance (especially *S. fallax*) in lawns, but no response to warming by *S. magellanicum* (Jassey et al., 2013). Subsequent measurements at this site showed variable responses of the two species to warming in wet and dry habitats (Buttler et al., 2015). Peat cores of lawns dominated by *Sphagnum fallax* that were transplanted to a warmer (+5°C) and drier location exhibited a 50% decline in *S. fallax* occurrence and the appearance of *S. magellanicum* after 3 years (Bragazza et al., 2016). These contrasting results suggest that the hummock‐hollow microtopography has a larger influence on *Sphagnum* responses to warming than species‐specific traits. Decline in *Sphagnum* growth (due to fertilization) at Mer Bleue bog in Ontario, Canada, favored *Polytrichum*, which is better adapted to dry conditions than *Sphagnum* species (Bubier, Moore, & Bledzki, 2007), but we saw no evidence of an increase in the presence of *Polytrichum* in our experiment. Warming experiments represent responses to “changed climate” rather than to “climate change” (Frolking et al., 2010) and do not necessarily measure changes in vegetation resulting from altered competition or succession (Turetsky et al., 2012). It may well be that a more gradual warming that allowed more time for replacement of one species by another would elicit a different result than what we observed after 3 years. However, losses in productivity in this experiment occurred across the entire range of warming treatments, losses were especially severe for *S. magellanicum*, a species characteristic of low hummocks that is more tolerant of desiccation than other *Sphagnum* species (Hájek & Beckett, 2008), and we saw no evidence of adjustments that would lead to a reversal of the loss.

Given its keystone role in the bog ecosystem, the decline of the *Sphagnum* community can be expected to have many follow‐on consequences to the structure and function of this and similar ecosystems as they are subjected to rising temperatures and increasing CO_2_ concentrations. Understanding how *Sphagnum* responds to climate change and associated changes in peatland hydrology will be critical to determining the fate of the C sink function of peatlands (Strack, Waddington, Lucchese, & Cagampan, 2009), but these evaluations must be done in the context of co‐occurring responses of other ecosystem components. Jassey et al. (2018) reported that there was a critical water table depth in their poor fen in Poland such that positive feedbacks among plants and fungi forced the system toward a critical tipping point as evaluated by ecosystem respiration. Kuiper, Mooij, Bragazza, and Robroek (2014) concluded from a species removal experiment that the *Sphagnum* community played the dominant mechanistic role in controlling C cycling, but the vascular plants in their experimental mesocosms controlled the shift of the ecosystem from C sink to C source during drought. One aspect of the regulatory role of *Sphagnum* in ecosystem function is through the production of polyphenols. Experimental warming of a peatland in the Jura mountains (France) caused a decrease in *Sphagnum* cover and altered the regulatory role of *Sphagnum* polyphenols on microbial community structure (Jassey et al., 2013), with potential effects on ecosystem C balance. In our study, warming caused a loss of 13–29 g C/m^2^ of NPP per °C temperature increase in elevated and ambient CO_2_, respectively. Some of this loss is likely to be compensated for by an increase in vascular plant NPP with warming and CO_2_ enrichment, especially if warming reduces the negative effect of mosses on vascular plant success (Jassey et al., 2013). Shrubs had the dominant influence on C sink strength in an ombrotrophic blanket bog in northern England (Ward et al., 2013), although in a transplant study (Bragazza et al., 2016), decreased *Sphagnum* productivity in elevated temperature was not compensated by enhanced vascular plant productivity.

Although compensatory C fluxes in response must be considered, the loss of *Sphagnum* productivity is nevertheless a substantial fraction of ecosystem NPP. Pretreatment NPP of trees and shrubs at the site was 187 g C/m^2^ (Griffiths et al., 2017), so *Sphagnum* NPP of 133 g C/m^2^ in +0°C enclosure in 2016 comprised 41% of total ecosystem NPP. Ongoing research at the site is determining all components of the C budget and will identify the effect of *Sphagnum* loss on overall NPP. Regardless of compensatory responses in NPP, however, peat accumulation generally declines as the fraction of productivity from moss declines (Frolking et al., 2010), so loss of *Sphagnum* could accelerate the reversal from carbon sink to source. Peatlands have been accumulating C for millennia because annual productivity exceeds annual decomposition, but modeling studies suggest that climatic warming will reverse this balance and become a positive feedback to the atmosphere by the end of the century (Gallego‐Sala et al., 2018). Shrubs and other vascular plants may determine tipping point between sink and source in peatlands, but the response of *Sphagnum* to warming will be a critical component of the integrated ecosystem response.

## CONFLICT OF INTEREST

None declared.

## AUTHOR CONTRIBUTIONS

R.J.N and J.C. developed the method and collected the data. R.J.N analyzed the data and wrote the manuscript. P.J.H. organized and operated the SPRUCE experiment. P.J.H and J.M.W. analyzed and interpreted the hydrology responses.

## Data Availability

Data are freely available from the SPRUCE project data archive: https://doi.org/10.25581/spruce.049/1426474
